# Successful Avoidance of Cicatricial Tracheobronchial Stenosis in a Patient With Endobronchial Tuberculosis by Early Administration of Systemic High-Dose Corticosteroids: A Case Report

**DOI:** 10.7759/cureus.60450

**Published:** 2024-05-16

**Authors:** Masami Yamazoe, Kento Furukawa, Kanami Nagano, Kazuya Takeda, Yutaro Nagano

**Affiliations:** 1 Respiratory Medicine, Hakodate Municipal Hospital, Hakodate, JPN; 2 Respiratory Medicine and Allergology, Sapporo Medical University School of Medicine, Sapporo, JPN

**Keywords:** tuberculosis, bronchoscopy, corticosteroid, cicatricial tracheobronchial stenosis, endobronchial tuberculosis

## Abstract

A 63-year-old Japanese woman was referred to our hospital due to dry cough, fever, hoarseness, stridor, and difficulty breathing. Chest computed tomography showed circumferential wall thickening in the trachea, carina, right main bronchus, and right upper lobe bronchus, and granular and nodular shadows in right S^2^. Flexible laryngofiberscopy showed yellowish dry respiratory secretions adhering to the subglottis. Bronchoscopic findings showed that the tracheobronchial mucosa was swollen, hyperemic, and covered with yellowish-white, cheese-like materials, and ulcerative lesions with white coatings were observed from the subglottis to the trachea, carina, right main bronchus, and right upper lobe bronchus. A diagnosis of endobronchial tuberculosis (EBTB) was confirmed by polymerase chain reaction testing, and cultures were positive for *Mycobacterium tuberculosis*. In addition to anti-tuberculosis chemotherapy, intravenous high-dose methylprednisolone reduced her severe respiratory symptoms and prevented cicatricial tracheobronchial stenosis. Early administration of systemic high-dose corticosteroids may be effective for EBTB patients with severely active tracheobronchial mucosal and submucosal lesions.

## Introduction

A tuberculous infection of the tracheobronchial tract is known as endobronchial tuberculosis (EBTB) [1]. Previously, EBTB was reported in 10-36.8% of patients with parenchymal tuberculous lesions or tuberculous lymphadenitis [2]. However, it has recently been reported that 54.3% of pulmonary tuberculosis patients have EBTB [3]. Patients with EBTB have a heterogeneous clinical course, and cicatricial tracheobronchial stenosis or obstruction that occurs during the healing process of EBTB is a significant complication of tuberculous airway lesions [4].

Treatment for active EBTB aims to eliminate *Mycobacterium tuberculosis* (*M. tuberculosis*) from the tracheobronchial tract and avoid cicatricial tracheobronchial stenosis, though stenosis can still develop even with prompt anti-tuberculosis chemotherapy [5].

The use of systemic corticosteroids to avoid cicatricial tracheobronchial stenosis in patients with EBTB is controversial.

This report highlights the efficacy of early administration of systemic high-dose corticosteroids to prevent the progression to cicatricial tracheobronchial stenosis in EBTB patients with severely active tracheobronchial mucosal and submucosal lesions.

## Case presentation

A 63-year-old Japanese woman started to experience a dry cough in September 2022. Her symptoms of a dry cough gradually worsened, and she was referred to our hospital in December 2022 due to fever, hoarseness, stridor, and difficulty breathing that had persisted since the previous day. She had no medical history and was not taking any medications. She was an ex-smoker (one pack per day for 20 years). Her husband developed pulmonary tuberculosis and was hospitalized for treatment one year after she got married at 28 years of age. On physical examination, she had a fever (38.4°C) and a low peripheral oxygen saturation of 96% on oxygen by nasal cannula at 2 L/min. Her height and weight were 157.5 cm and 50.9 kg, respectively. Her blood pressure, heart rate, and respiratory rate were 136/88 mmHg, 110 beats/min, and 21 breaths/min, respectively. Stridor was audible in the neck during auscultation.

**Figure 1:**
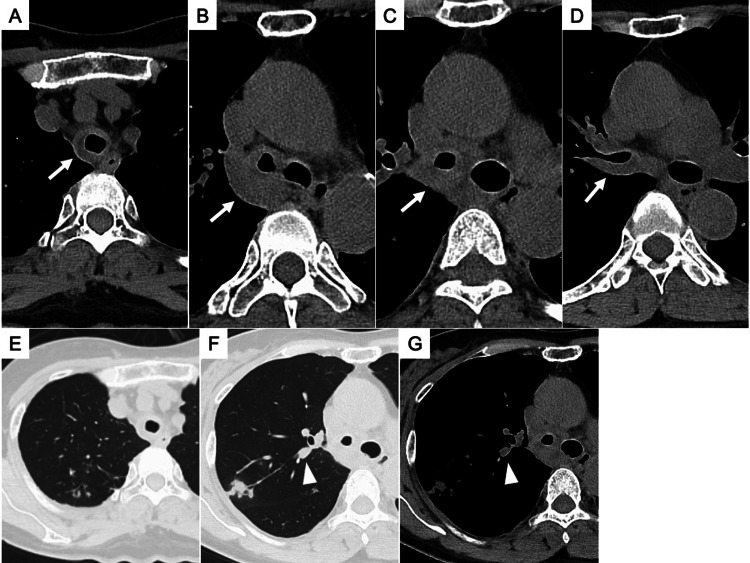
Chest computed tomography findings at the initial presentation. Chest computed tomography at the initial presentation shows circumferential wall thickening (white arrow) in the trachea (A), carina (B), right main bronchus (C), and right upper lobe bronchus (D). Granular and nodular shadows in the right S^2^ of the lung (E, F), and a nodular shadow that appears to be mucoid impaction in the orifice of the right B^2^ (white arrowhead) are also seen (F, G).

**Figure 2:**
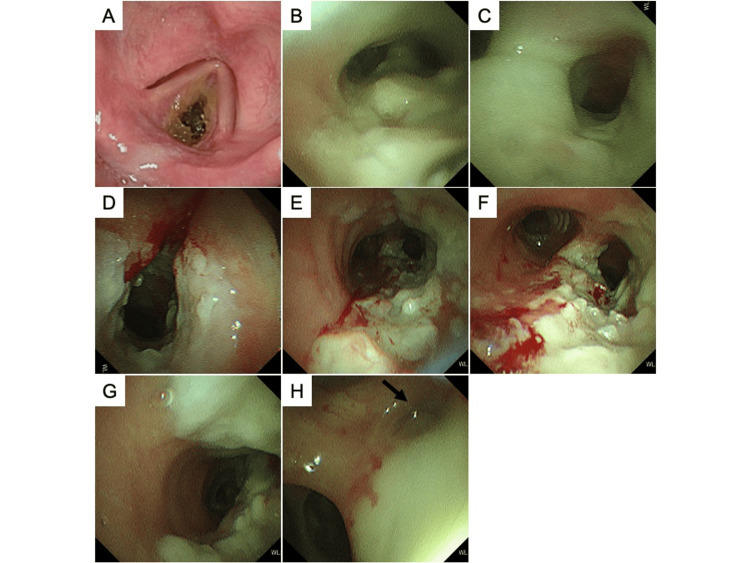
The findings of flexible laryngofiberscopy and bronchoscopy at the initial presentation. Flexible laryngofiberscopy shows yellowish, dry, respiratory secretions adhering to the subglottis (A). Bronchoscopy shows that the yellowish-white, cheese-like materials are found circumferentially from the trachea to the carina (B) and the right main bronchus (C). After removing the yellowish-white, cheese-like materials, the tracheobronchial mucosa appears swollen and hyperemic, and ulcerative lesions with white coatings are observed from the subglottis (D) to the trachea (E), carina (F), right main bronchus (G), and right upper lobe bronchus (H). The orifice of the right B^2^ (black arrow) is obstructed by yellowish-white, cheese-like materials (H).

Her blood tests on admission showed the following: white blood cells = 11,900/μL (neutrophils = 84.6%; lymphocytes = 9.3%; monocytes = 5.2%; eosinophils = 0.6%; basophils = 0.3%); hemoglobin = 11.1 g/dL; platelets = 36.5x10^4^/μL; C-reactive protein = 4.84 mg/dL (normal ≦ 0.3 mg/dL); procalcitonin = 0.08 ng/mL (normal < 0.50 ng/mL); and glycosylated hemoglobin (HbA1c) = 5.9% (normal = 4.6-6.2%). Renal and liver functions were normal. The result of QuantiFERON-TB Gold Plus testing was positive. On arterial blood gas analysis, the partial pressure of arterial oxygen was 139 mmHg, the partial pressure of arterial carbon dioxide was 40.2 mmHg, and pH was 7.377 on oxygen by nasal cannula at 2 L/min. A chest X-ray showed nodular shadows in the right upper lung field. Computed tomography (CT) of the chest showed circumferential wall thickening in the trachea, carina, right main bronchus, and right upper lobe bronchus (Figures 1A-1D). Granular and nodular shadows in the right S^2^ of the lung, and a nodular shadow that appeared to be mucoid impaction in the orifice of right B^2^ were also seen (Figures 1E-1G). No obvious abnormalities were seen on the neck CT. Continuous nebulized beta-2 agonists did not improve her stridor, and there was no sputum expectoration. Flexible laryngofiberscopy showed yellowish dry respiratory secretions adhering to the subglottis (Figure 2A). After nasal intubation with an endotracheal tube (internal diameter of 5.0 mm; Portex Tracheal Tube®︎, Smiths Medical Japan Ltd., Tokyo, Japan) using a thin bronchoscope (BF-P290, Olympus Corporation, Tokyo, Japan), bronchoscopy was performed to investigate the cause of the tracheal and bronchial wall thickening. Yellowish-white, cheese-like materials were found circumferentially from the trachea to the carina and right main bronchus (Figures 2B, 2C). On pathological examination, these materials showed fibrin exudates consisting of neutrophils and fibrin deposits. The fibrin exudates were removed as much as possible with forceps and suction to relieve tracheobronchial stenosis. The tracheobronchial mucosa was seen to be swollen and hyperemic, and ulcerative lesions with the mucosa covered by a white coating were observed from the subglottis to the trachea, carina, right main bronchus, and right upper lobe bronchus (Figures 2D-2H). The orifice of the right B^2^ was obstructed by fibrin exudates (Figure 2H). Although staining for acid-fast bacilli (AFB) was negative, the bronchial aspirate was positive on polymerase chain reaction (PCR) testing and cultures for *M. tuberculosis*. The fibrin exudates also showed that AFB staining was negative, but the cultures for *M. tuberculosis* were positive. Routine bacterial cultures of the bronchial aspirate and fibrin exudates were negative. Therefore, the diagnosis was EBTB, and the patient was admitted to our hospital. Her stridor improved after completing the bronchoscopy while removing the nasal intubation tube.

On the next day of admission (day two), anti-tuberculosis chemotherapy with isoniazid (INH; 250 mg/day), rifampicin (RFP; 450 mg/day), ethambutol (EB; 750 mg/day), and pyrazinamide (PZA; 1250 mg/day) was started. In addition, intravenous high-dose methylprednisolone (mPSL; 125 mg/day (2 mg/kg/day)) was administered to prevent progression to cicatricial tracheobronchial stenosis on day four. The difficulty breathing subsided, and she was withdrawn from oxygen therapy by nasal cannula on day four. The fever and cough subsided on day five, and the hoarseness improved on day six. The dose of intravenous mPSL was reduced to 62.5 mg/day (1 mg/kg/day) on day 11 and was continued for two weeks. The intravenous mPSL was then replaced with oral prednisolone (PSL) at a dose of 30 mg/day. The dose of PSL was then gradually tapered. On day seven, the first sputum examination showed negative AFB staining, but cultures for *M. tuberculosis* were positive. On day 14, the second sputum examination showed that AFB staining and cultures for *M. tuberculosis* were positive. On day 35, the fifth sputum examination showed that AFB staining and cultures for *M. tuberculosis* were negative. Subsequent sputum examinations for AFB staining and cultures for *M. tuberculosis* continued to be negative. No drug resistance was detected on drug susceptibility testing for *M. tuberculosis*. The patient was discharged home on day 52.

**Figure 3:**
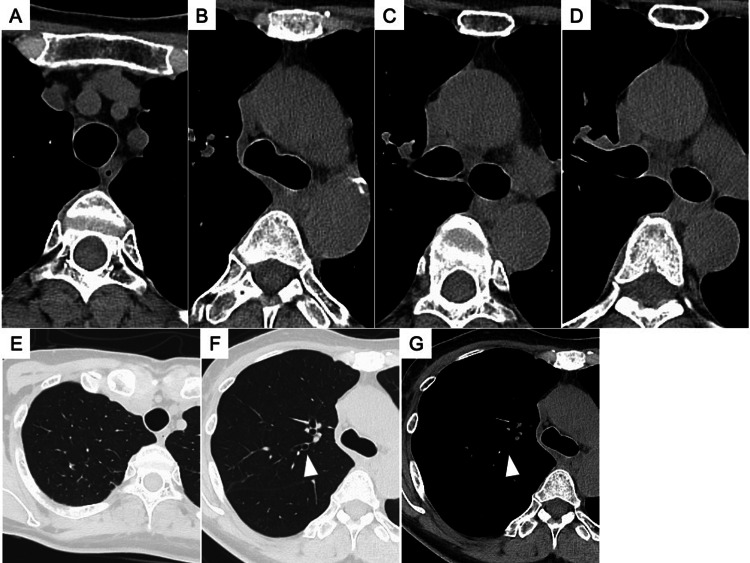
Chest computed tomography findings at the end of anti-tuberculosis treatment. Chest computed tomography at the end of anti-tuberculosis treatment shows that the circumferential wall thickening in the trachea (A), carina (B), right main bronchus (C), and right upper lobe bronchus (D) has improved. Granular and nodular shadows in the right S^2^ of the lung and a nodular shadow in the orifice of the right B^2^ (white arrowhead) have also improved (E-G).

**Figure 4:**
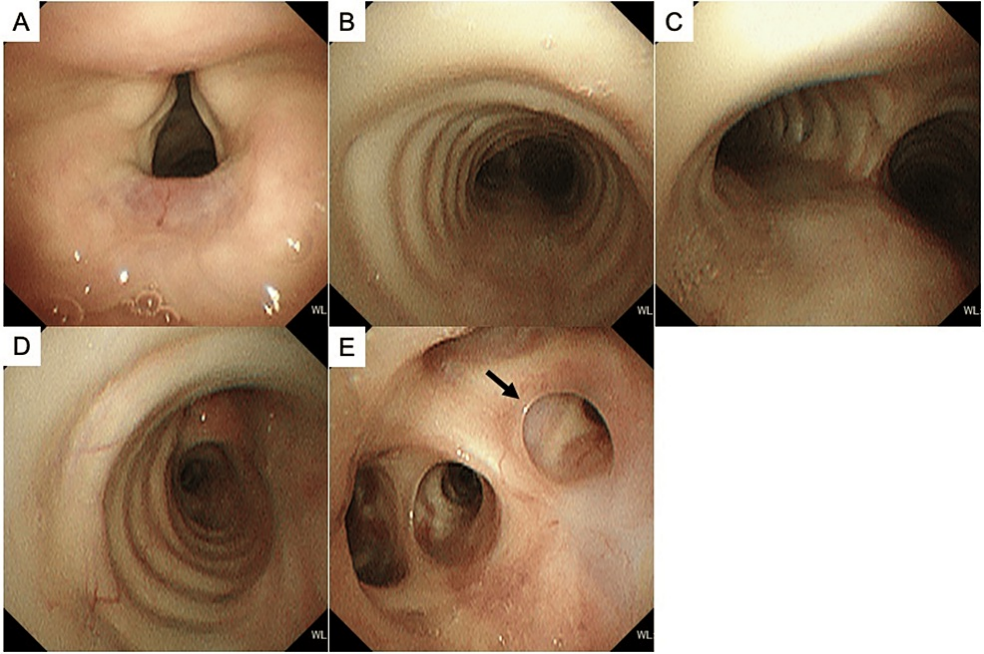
The findings of bronchoscopy at the end of anti-tuberculosis treatment. Bronchoscopy at the end of anti-tuberculosis treatment shows that ulcerative lesions with white coatings have disappeared, and no cicatricial tracheobronchial stenosis is observed from the subglottis (A) to the trachea (B), carina (C), right main bronchus (D), and right upper lobe bronchus (E). No occlusions of the orifice of the right B^2^ (black arrow) are observed (E).

Two months after the start of anti-tuberculosis chemotherapy, the treatment with INH and RFP was continued for seven months. The oral PSL was gradually tapered and discontinued three months after intravenous mPSL administration. The oral trimethoprim-sulfamethoxazole was administered to prevent *Pneumocystis jirovecii* pneumonia while receiving systemic corticosteroids. Her respiratory status remained stable during hospitalization and after discharge, with no adverse events. Nine months after starting anti-tuberculosis treatment, chest CT showed that the circumferential wall thickening in the trachea, carina, right main bronchus, and right upper lobe bronchus had improved (Figures 3A-3D). Granular and nodular shadows in right S^2^ and a nodular shadow in the orifice of right B^2^ had also improved (Figures 3E-3G). On bronchoscopy, ulcerative lesions with white coatings had disappeared, and no cicatricial tracheobronchial stenosis was observed (Figure 4). Twelve months after the discontinuation of anti-tuberculosis chemotherapy, there were no respiratory symptoms or abnormalities on the chest CT suggestive of EBTB recurrence.

## Discussion

The bronchoscopic findings of EBTB have a strong relationship to the pathological changes and can be divided into seven subtypes: (I) nonspecific bronchitic subtype; (II) edematous-hyperemic subtype; (III) actively caseating subtype; (IV) granular subtype; (V) ulcerative subtype; (VI) tumorous subtype; and (VII) fibrostenotic subtype [6]. Edematous-hyperemic, actively caseating, and granular subtypes are the most frequently seen among these seven subtypes; however, multiple subtypes often coexist in the same individual [7]. In the early stages of EBTB, pathological changes begin with localized mucosal and submucosal edema-hyperemia due to the infiltration of inflammatory cells, mainly lymphocytes. Subsequently, tubercular nodules develop as prominent pathological changes. As the disease progresses further, caseous necrosis appears in the tubercular nodules, which may cause the mucosa to ulcerate. Mucosal ulcers may progress into the tracheobronchial wall and become deeper, resulting in the formation of polyps toward the bronchial lumen. In the advanced stages, fibrous hyperplasia and contractures develop and cause the tracheobronchial stenosis [1,7]. In the present case, the bronchoscopic findings before the start of treatment showed the coexistence of actively caseating and ulcerative subtypes extending from the subglottis to the trachea, carina, right main bronchus, and right upper lobe bronchus.

The pathogenesis of EBTB has been linked to the following: (I) direct infiltration of disease from the lungs; (II) infected sputum/secretions causing direct implantation of organisms; (III) hematogenous spread; (IV) lymphatic dissemination; and (V) erosion of lymph nodes into the trachea or bronchus [4]. In the present case, based on the chest CT and bronchoscopic findings, the pathogenesis of EBTB was presumed to be mucosal infiltration of tuberculosis foci in lymphatic dissemination or continuous progression from the right S^2^ peripheral lesions of the lung.

In patients with EBTB, cicatricial tracheobronchial stenosis often remains even after appropriate anti-tuberculosis chemotherapy, and complications of symptoms such as wheezing and coughing, atelectasis, and obstructive pneumonia have been noted [5,8,9]. Therefore, in addition to anti-tuberculosis chemotherapy, the need for treatment to prevent progression to cicatricial tracheobronchial stenosis is considered in the treatment of EBTB [1]. Kurasawa et al. reported that lesions with a transverse extension greater than half the circumference of the bronchus at the initial bronchoscopy were likely to develop later cicatricial tracheobronchial stenosis or obstruction [10]. Arai reported that in the follow-up of bronchoscopic findings of EBTB, cicatricial tracheobronchial stenosis was likely to occur in cases where the lesion was circumferential or reached the submucosa [11]. Um et al. reported that the independent predictors contributing to persistent tracheobronchial stenosis following anti-tuberculosis chemotherapy in patients with EBTB were age > 45 years, pure or combined fibrostenotic subtype, and duration of chief complaints before anti-tuberculosis chemotherapy > 90 days [5]. Jung et al. reported that the risk factors for developing cicatricial tracheobronchial stenosis after treatment for EBTB included a decreased forced expiratory volume in one second and the total length of lesions (>20 mm) at the start of treatment [3]. Taking these factors into consideration implies that delayed diagnosis of EBTB is likely to result in disease progression and cicatricial tracheobronchial stenosis. In the present case, the patient was over 45 years of age, and more than 90 days had elapsed since the onset of the chief complaint to the initiation of anti-tuberculosis chemotherapy. Moreover, the bronchoscopic findings before starting treatment showed the coexistence of actively caseating and ulcerative subtypes, and these lesions spread circumferentially. It was expected that cicatricial tracheobronchial stenosis would progress if preventive treatment in addition to anti-tuberculosis chemotherapy was not performed.

To avoid cicatricial tracheobronchial stenosis in adult patients with EBTB, adjuvant therapies such as INH inhalation [12], streptomycin plus corticosteroid inhalation [13], local steroid spray under bronchoscopy [14], and systemic corticosteroids [15-17] have been attempted. However, methods to prevent progression to cicatricial tracheobronchial stenosis and obstruction during the healing process of EBTB have not yet been established. Of these adjuvant therapies, there are reports that administration of systemic corticosteroids can avoid cicatricial tracheobronchial stenosis [15,17] and one negative report on their effectiveness [16]. Mo et al. prospectively evaluated the effects of PSL 40 mg/day for four weeks within the first month of anti-tuberculosis chemotherapy [15]. They suggested that, in the early period of anti-tuberculosis chemotherapy, corticosteroids were effective in the treatment of tracheobronchial stenosis when EBTB was the edematous type, not the infiltrative type. Ikeno et al. reported that, in addition to anti-tuberculosis chemotherapy, administration of mPSL with a dose of 2 mg/kg/day for two weeks, followed by tapering every two weeks and discontinuation, could avoid cicatricial tracheobronchial stenosis for an EBTB patient with the coexistence of granular and ulcerative subtypes extending from the subglottis to the trachea, carina, and bilateral main bronchi [17]. In contrast, Park et al. prospectively evaluated the effects of PSL with a dose of 0.5-1.0 mg/kg/day for six to eight weeks, followed by tapering off for four to eight weeks in the treatment of EBTB [16]. They found that the healing rate of bronchoscopic findings and changes in pulmonary function showed no significant differences between the anti-tuberculosis chemotherapy only and the combined anti-tuberculosis chemotherapy with PSL groups. The difference in the effects of administration of systemic corticosteroids in the treatment of EBTB may be due to varied dose schedules of corticosteroids. As demonstrated in the present case, early administration of systemic high-dose corticosteroids may be effective in preventing progression to cicatricial tracheobronchial stenosis.

## Conclusions

A case of EBTB with severely active tracheobronchial mucosal and submucosal lesions was described. Early administration of systemic high-dose corticosteroids as adjuvant therapy to anti-tuberculous chemotherapy reduced symptoms and prevented cicatricial tracheobronchial stenosis. In advanced cases of EBTB presenting with ulcerative lesions, severe cicatricial tracheobronchial stenosis may occur during the healing process, which may lead to serious complications and sequelae. In addition to anti-tuberculosis chemotherapy, treatment to prevent cicatricial tracheobronchial stenosis is required, and early administration of systemic high-dose corticosteroids may be effective for EBTB patients with severely active tracheobronchial mucosal and submucosal lesions. Further study is expected to help elucidate the target subtype for corticosteroids, as well as the optimal timing, dose, and duration of corticosteroid administration.
